# Long-Term Ultrasonography Follow-Up of Thyroid Colloid Cysts at the Health Center: A Single-Center Study

**DOI:** 10.1155/2015/324581

**Published:** 2015-10-22

**Authors:** Myung Ho Rho, Dong Wook Kim

**Affiliations:** ^1^Department of Radiology, Kangbuk Samsung Hospital, Sungkyunkwan University School of Medicine, Seoul 110-746, Republic of Korea; ^2^Department of Radiology, Busan Paik Hospital, Inje University School of Medicine, Busan 614-734, Republic of Korea

## Abstract

*Objective*. No previous study has employed long-term follow-up ultrasonography (US) examinations for evaluating thyroid colloid cysts (TCCs) in the general population. This study aimed to assess the interval changes of TCCs at the health center by evaluating long-term US follow-up examinations. *Methods*. For evaluation of the thyroid gland at our health center from 2006 to 2010, 3692 individuals underwent 4 or more thyroid US examinations at an interval of 1 year or 2 years. We assessed the interval changes of TCCs ≥ 5 mm on US follow-up examinations. *Results*. Of the 3692 subjects, only 115 (3.1%) showed TCCs ≥ 5 mm on one or more thyroid US examinations. The interval changes in TCCs, as shown by the thyroid US examinations performed during the study period, were classified as follows: no interval change (*n* = 60), gradual increase (*n* = 37), gradual decrease (*n* = 6), positive fluctuation (*n* = 10), negative fluctuation (*n* = 0), and disappearance (*n* = 2). No subject reported any relevant symptom pertaining to TCCs. *Conclusions*. Overall, follow-up US examinations showed various interval changes in TCCs, but a majority of TCCs showed no interval change or a gradual increase in size.

## 1. Introduction

A thyroid nodule is a discrete lesion within the thyroid gland that is distinguishable from the adjacent thyroid parenchyma by ultrasonography (US) examination [1]. Thyroid nodules are present in approximately 50% of adults and increase in prevalence with age [2]. The majority of thyroid nodules are benign and asymptomatic, which are not a cause for concern [1, 2] However, the minority of thyroid nodules show suspicious sonographic features, and thus US-guided fine-needle aspiration has been performed [3–5]. A thyroid colloid cyst (TCC) is a common benign thyroid nodule, which shows marked follicular dilatation, epithelial flattening, and a dense viscous material comprising a concentrated solution of thyroglobulin on histological analysis [6]. In the general population, a TCC is commonly detected by chance on thyroid US; however, its clinical significance is low because a TCC is not associated with a thyroid malignancy and is easily diagnosed by US [7]. The typical sonographic feature of a TCC is an anechoic cyst with comet-tail artifacts [7]. However, a TCC can result in a symptomatic attack because of intracystic hemorrhage or a gradual increase in size [8]. A recent study of patients who underwent a lobectomy for the treatment of papillary thyroid microcarcinoma demonstrated that TCCs have various interval changes [9]. However, no data exists regarding the long-term interval changes of TCCs in the general population.

To the best of our knowledge, no previous study has employed long-term follow-up US examinations for evaluating TCCs in the general population. This study assessed the prevalence and interval changes of TCCs ≥ 5 mm at their largest diameter in subjects who received 4 or more thyroid US examinations at our health center.

## 2. Materials and Methods

### 2.1. Subjects

From January 2006 to December 2010, many individuals underwent one or more thyroid US examinations at the Health Center of Kangbuk Samsung Hospital. Among them, those who underwent 4 or more thyroid US examinations at 1- or 2-year interval were selected for this study. In addition, individuals who had had 3 or less US follow-up examinations were excluded from this study. Ultimately, 3692 subjects (2670 women and 1021 men; age range: 21–78 years; mean age: 44.4 ± 8.0 years) were included in this study. The institutional review board approved this retrospective study (IRB KBC14073), and informed consent was not required for the review of medical records and US images.

### 2.2. Thyroid Ultrasonography and Sonographic Classification

Thyroid US was performed by one of six radiologists using a high-resolution ultrasound instrument (Logiq E9; GE Medical Systems, Milwaukee, WI, USA), equipped with a 5–15 MHz linear probe. A TCC was defined as a pure thyroid cyst with intracystic comet-tail artifact(s). In the sonographic measurement of a TCC, its largest diameter was selected regardless of the anteroposterior, the transverse, or the longitudinal diameter. To clarify the evaluation of an interval change for the TCCs, individuals with a TCC ≥ 5 mm at their largest diameter on any follow-up US examination were enrolled in this study. In the case subjects with multiple TCCs ≥ 5 mm, only the largest TCC was evaluated in this study. Follow-up US examinations were performed at the Health Center of Kangbuk Samsung Hospital in the following manner: after the initial thyroid US, a follow-up US examination was performed after an interval of 1 year or 2 years, depending on the subject's preference.

On the basis of the US follow-up examinations, each TCC was retrospectively classified into 1 of 6 diagnostic categories by a single radiologist: (1) no interval change, when an interval change of ±10% occurred in the TCC at its largest diameter; (2) gradual increase, when an interval increase of >10% at the largest diameter of the TCC occurred but did not fluctuate during the subsequent US follow-up examinations; (3) gradual decrease, when an interval decrease of >10% at the largest diameter of the TCC occurred but did not fluctuate during the subsequent US follow-up examinations; (4) positive fluctuation, when an interval decrease occurred in the TCC during the early US follow-up examinations and an interval increase occurred in the TCC during the late US follow-up examinations; (5) negative fluctuation, when an interval increase occurred in the TCC during the early US follow-up examinations and an interval decrease occurred in the TCC during the later US follow-up examinations; and (6) disappearance, when no TCC was visualized during the US follow-up examinations.

### 2.3. Serological Evaluation

In all the subjects, serum concentrations of free T3 (reference range [RR], 2.0–4.4 pg/mL), free T4 (RR, 0.7–2.0 ng/dL), and thyroid-stimulating hormone (TSH; RR, 0.27–4.2 *μ*IU/mL) were evaluated on the same day as the thyroid US examination. All serological parameters were evaluated using an electrochemiluminescence immunoassay on the Elecsys automatic system (Roche Diagnostics Deutschland GmbH, Mannheim, Germany).

## 3. Results

**Figure 1:**
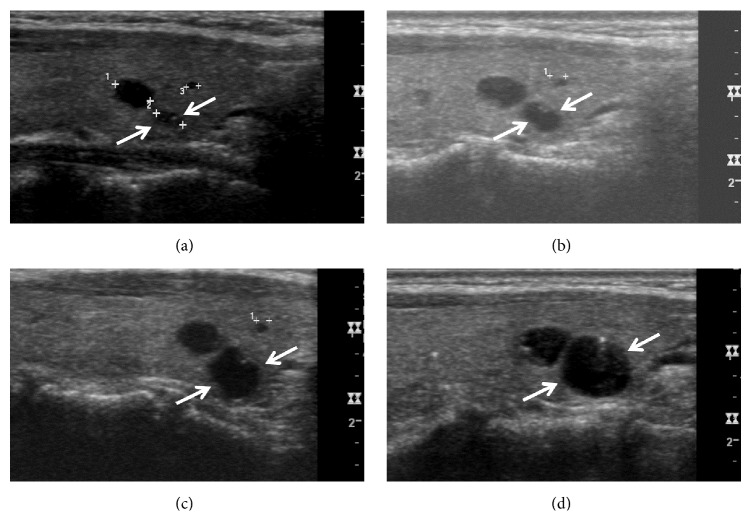
A thyroid colloid cyst with gradual increase in a 50-year-old woman with gradual increase. Longitudinal sonogram (a) shows a colloid cyst with an anechoic nodule and a comet-tail artifact in the right thyroid lobe (arrows, 3.3 mm at its largest diameter). Upon sequential US follow-ups, this colloid cyst demonstrates a gradual increase at a 1-year (b) (arrows, 4.5 mm at its largest diameter), a 2-year (c) (arrows, 6.3 mm at its largest diameter), and a 3-year (d) (arrows, 8.2 mm at its largest diameter) US follow-ups.

**Figure 2:**
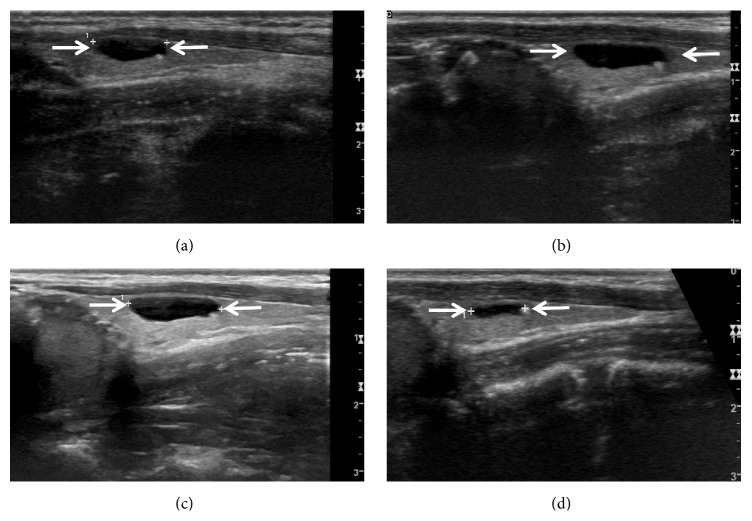
A thyroid colloid cyst with positive fluctuation in a 40-year-old woman. Longitudinal sonogram (a) shows a colloid cyst with an anechoic nodule and a comet-tail artifact in the left thyroid lobe (arrows, 11.1 mm at its largest diameter). Upon sequential US follow-ups, this colloid cyst demonstrates a gradual increase at a 1-year (b) (arrows, 13.0 mm at its largest diameter) and a 2-year (c) (arrows, 13.8 mm at its largest diameter) US follow-ups, but it shows a moderate decrease on 3-year follow-up US (d) (arrows, 8.4 mm at its largest diameter).

Of the 3692 subjects, 115 (99 women and 16 men; age range: 21–71 years; mean age: 45.2 ± 8.3 years) had TCCs ≥ 5 mm at the largest diameter of the TCCs (range: 5.0–32.0 mm; mean: 8.6 ± 4.0 mm). Based on the long-term US follow-up examinations (range: 47–88 months and 4–7 sessions; mean: 58.4 ± 11.5 months and  4.7 ± 1.0 sessions), the dominant TCCs were classified according to interval changes: no interval change (*n* = 60), gradual increase (*n* = 37), gradual decrease (*n* = 6), positive fluctuation (*n* = 10), negative fluctuation (*n* = 0), and disappearance (*n* = 2) (Figures 1 and 2). The results of the long-term follow-up US examinations of 115 cases of TCCs are summarized in Table 1.

In 36 of the 115 subjects, follow-up US examinations revealed a TCC ≥ 10 mm at its largest diameter. During the follow-up US examinations, only one case showed an increase that was 2 or more times its largest diameter (from 12 mm to 32 mm), but it did not result in a symptomatic attack. Neither did the subjects of this study report any relevant symptoms nor did they undergo fine-needle aspiration or ethanol ablation for management of the TCCs.

The initial laboratory findings of free T3 (mean: 3.13 ± 0.28 pg/mL; range: 2.27–4.1 pg/mL), free T4 (mean: 1.25 ± 0.13 ng/dL; range: 0.97–1.59 ng/dL), and thyroid-stimulating hormone (mean: 2.29 ± 1.35 *μ*IU/mL; range: 0.41–8.77 *μ*IU/mL) indicated euthyroid (*n* = 107) and subclinical hypothyroidism (*n* = 8). No subject underwent thyroid hormone therapy. Among the 8 subjects with subclinical hypothyroidism, 4 showed normal values on follow-up thyroid function tests.

## 4. Discussion

High-resolution thyroid US has been globally used for the initial evaluation of thyroid nodules, and several sonographic features that suggest benign and malignant thyroid nodules have been established [1, 5, 10]. A recent study has emphasized that recognition of specific morphologic patterns, such as spongiform configuration, cyst with colloid clot, giraffe pattern, and diffuse hyperechogenicity, is an accurate method of identifying benign thyroid nodules that do not require cytologic evaluation [11]. In particular, a TCC is a well-known benign thyroid nodule since the clinical significance of the comet-tail artifact in thyroid US has been emphasized [7]. However, the size of a TCC may gradually increase or it may result in a symptomatic attack by intralesional hemorrhage.

To date, only one study regarding the long-term US follow-up of TCCs has been performed [9]. In that study, US follow-up examinations were conducted in 35 patients who underwent a lobectomy for the treatment of papillary thyroid microcarcinoma; the US findings of the 35 TCCs ≥ 3 mm at the largest diameter included no interval change (22.9%, 8/35), gradual increase (22.9%, 8/35), gradual decrease (14.3%, 5/35), positive fluctuation (8.6% 3/35), negative fluctuation (17.1%, 6/35), disappearance (14.3%, 5/35), and new detection (17.1%, 6/35). In the present study, most TCCs showed no interval change (52.2%, 60/115) or a gradual increase (32.2%, 37/115). The exact reason for the discrepant results is unclear but may be attributed to the following differences between the two studies. First, the present study included only those individuals who had no history of thyroid surgery and a normal thyroid function, whereas the previous study only included patients who were undergoing thyroid hormone therapy after a thyroid surgery. Second, the size criteria of TCCs were different between the two studies.

Symptomatic TCCs can be associated with an intralesional hemorrhage or a significant increase in the volume [2, 8]. In the present study, none of TCC cases involved a symptomatic attack. Furthermore, the participant with the largest TCC (largest diameter: 32 mm) showed no subjective symptoms, probably due to the longitudinal orientation of the TCC. Therefore, we believe that US follow-up examinations for asymptomatic TCCs are unnecessary. However, for the management of symptomatic TCCs, US-guided fine-needle aspiration or ethanol ablation can be considered [4, 8].

This study has several limitations. First, only a single dominant TCC in each subject was investigated. Thus, many nondominant TCC cases ≥ 5 mm at their largest diameter could have been excluded from the study. This could have resulted in the low prevalence of TCC ≥ 5 mm at their largest diameter (3.1%, 115/3692) in our study population. Second, the US examinations were performed by six radiologists. Interobserver variability should thus be considered. In the previous study [12], the interobserver variation for sonographic measurement of thyroid nodules is approximately 50%. Third, a TCC without intracystic comet-tail artifact would be excluded. Fourth, owing to the retrospective nature of this study, we used the largest diameter rather than the volume for the sonographic follow-up of a TCC. Finally, the follow-up periods were not uniform between the various subjects.

In conclusion, various interval changes were observed in the TCCs; however, most TCCs showed no interval change or a gradual increase in size, without an abrupt increase.

## Figures and Tables

**Table 1:** The sonographic follow-up results of 115 subjects with thyroid colloid cysts.

Interval change	Gender (women : men)	Age (years)	Location	Mean size (mm)
No interval change (60)	50 : 10	43.7 ± 7.1 (28–71)	Rt (31), Lt (28), Isth (1)	7.7 ± 2.8 (5–17)
Gradual increase (37)	33 : 4	46.9 ± 9.9 (26–66)	Rt (21), Lt (15), Isth (1)	10.2 ± 5.2 (5–32)
Gradual decrease (6)	6 : 0	44.8 ± 5.9 (39–55)	Rt (4), Lt (2), Isth (0)	7.6 ± 2.2 (5–10)
Positive fluctuation (10)	9 : 1	45.0 ± 7.1 (34–56)	Rt (3), Lt (6), Isth (1)	9.1 ± 4.1 (5–18)
Negative fluctuation (0)	0		0	0
Disappearance (2)	1 : 1	60.5 ± 13.4 (51–70)	Rt (1), Lt (1), Isth (0)	5.5 ± 0.7 (5-6)

*Note*. Data presented in parentheses are number of each item. Rt: right; Lt: left.
